# Impact of Distinct Antiandrogen Exposures on the Plasma Metabolome in Feminizing Gender-affirming Hormone Therapy

**DOI:** 10.1210/clinem/dgae226

**Published:** 2024-04-13

**Authors:** Rebecca Shepherd, Lachlan M Angus, Toby Mansell, Bridget Arman, Bo Won Kim, Katherine Lange, David Burgner, Jessica A Kerr, Ken Pang, Jeffrey D Zajac, Richard Saffery, Ada Cheung, Boris Novakovic

**Affiliations:** Molecular Immunity, Infection and Immunity Theme, Murdoch Children's Research Institute, Royal Children's Hospital, Parkville, VIC 3052, Australia; Department of Medicine (Austin Health), The University of Melbourne, Parkville, VIC 3052, Australia; Department of Endocrinology, Austin Health, Heidelberg, VIC 3084, Australia; Department of Paediatrics, The University of Melbourne, Parkville, VIC 3052, Australia; Inflammatory Origins, Infection and Immunity Theme, Murdoch Children's Research Institute, Royal Children's Hospital, Parkville, VIC 3052, Australia; Therapeutics Discovery and Vascular Function Group, Department of Obstetrics and Gynaecology, University of Melbourne, Mercy Hospital for Women, Heidelberg, VIC 3084, Australia; Mercy Perinatal, Mercy Hospital for Women, Heidelberg, VIC 3084, Australia; Department of Obstetrics and Gynaecology, University of Melbourne, Mercy Hospital for Women, Heidelberg, VIC 3084, Australia; Molecular Immunity, Infection and Immunity Theme, Murdoch Children's Research Institute, Royal Children's Hospital, Parkville, VIC 3052, Australia; Department of Paediatrics, The University of Melbourne, Parkville, VIC 3052, Australia; Department of Paediatrics, The University of Melbourne, Parkville, VIC 3052, Australia; The Centre for Community Child Health, Murdoch Children's Research Institute, Royal Children's Hospital, Parkville, VIC 3052, Australia; Department of Paediatrics, The University of Melbourne, Parkville, VIC 3052, Australia; Inflammatory Origins, Infection and Immunity Theme, Murdoch Children's Research Institute, Royal Children's Hospital, Parkville, VIC 3052, Australia; Department of Paediatrics, The University of Melbourne, Parkville, VIC 3052, Australia; Department of Psychological Medicine, University of Otago, Christchurch 8011, New Zealand; Murdoch Children's Research Institute, Centre for Adolescent Health, Population Health Theme, Parkville, VIC 3052, Australia; Department of Paediatrics, University of Melbourne, Parkville, VIC 3052, Australia; Brain and Mind Research, Murdoch Children's Research Institute, Royal Children's Hospital, Parkville, VIC 3052, Australia; Department of Adolescent Medicine, Royal Children's Hospital, Parkville, VIC 3052, Australia; Department of Medicine (Austin Health), The University of Melbourne, Parkville, VIC 3052, Australia; Department of Endocrinology, Austin Health, Heidelberg, VIC 3084, Australia; Molecular Immunity, Infection and Immunity Theme, Murdoch Children's Research Institute, Royal Children's Hospital, Parkville, VIC 3052, Australia; Department of Paediatrics, The University of Melbourne, Parkville, VIC 3052, Australia; Department of Medicine (Austin Health), The University of Melbourne, Parkville, VIC 3052, Australia; Department of Endocrinology, Austin Health, Heidelberg, VIC 3084, Australia; Molecular Immunity, Infection and Immunity Theme, Murdoch Children's Research Institute, Royal Children's Hospital, Parkville, VIC 3052, Australia; Department of Paediatrics, The University of Melbourne, Parkville, VIC 3052, Australia

**Keywords:** transgender, GAHT, sex hormones, metabolome, metabolism, estradiol

## Abstract

**Context:**

The plasma metabolome is a functional readout of metabolic activity and is associated with phenotypes exhibiting sexual dimorphism, such as cardiovascular disease. Sex hormones are thought to play a key role in driving sexual dimorphism.

**Objective:**

Gender-affirming hormone therapy (GAHT) is a cornerstone of transgender care, but longitudinal changes in the plasma metabolome with feminizing GAHT have not been described.

**Methods:**

Blood samples were collected at baseline and after 3 and 6 months of GAHT from transgender women (n = 53). Participants were randomized to different anti-androgens, cyproterone acetate or spironolactone. Nuclear magnetic resonance-based metabolomics was used to measure 249 metabolic biomarkers in plasma. Additionally, we used metabolic biomarker data from an unrelated cohort of children and their parents (n = 3748) to identify sex- and age-related metabolite patterns.

**Results:**

We identified 43 metabolic biomarkers altered after 6 months in both anti-androgen groups, most belonging to the very low- or low-density lipoprotein subclasses, with all but 1 showing a decrease. We observed a cyproterone acetate-specific decrease in glutamine, glycine, and alanine levels. Notably, of the metabolic biomarkers exhibiting the most abundant “sex- and age-related” pattern (higher in assigned female children and lower in assigned female adults, relative to assigned males), 80% were significantly lowered after GAHT, reflecting a shift toward the adult female profile.

**Conclusion:**

Our results suggest an anti-atherogenic signature in the plasma metabolome after the first 6 months of feminizing GAHT, with cyproterone acetate also reducing specific plasma amino acids. This study provides novel insight into the metabolic changes occurring across feminizing GAHT.

Gender incongruence occurs when an individual's gender identity and sex assigned at birth do not align. Many, but not all, transgender individuals undergo feminization or masculinization through gender-affirming hormone therapy (GAHT) (1). GAHT aims to align physical characteristics with an individual’s gender identity and improves emotional well-being and social functioning within the first few months of treatment (1-5). However, the metabolic impacts of GAHT are understudied.

Anti-androgens such as spironolactone and cyproterone acetate are commonly used alongside estradiol in feminizing GAHT to reduce testosterone levels, but few studies have compared the effects of these anti-androgens on metabolic biomarkers. Spironolactone is a mineralocorticoid antagonist and acts as an anti-androgen via partial androgen receptor antagonization, weak inhibition of testosterone biosynthesis enzymes (17α-hydrolase and 17,20-lyase), and weak progestogenic and estrogenic activity thought to suppress GnRH and gonadotropin release from the brain, which subsequently lowers testosterone (6). Cyproterone acetate is a potent anti-androgen that acts through its competitive antagonization of peripheral androgen receptor sites and activation of the progesterone receptor (6).

There is a well-established sexual dimorphism in circulating metabolite profiles, with cisgender male and cisgender female adults exhibiting differences in their circulating metabolomes, with sex hormones thought to explain some of these differences. Sex-associated levels of high-density (HDL) and low-density (LDL) lipoproteins have been consistently reported, with premenopausal cisgender females showing higher HDL measures and lower LDL measures than age-matched cisgender males or postmenopausal cisgender females (7). Moreover, sex-associated levels of atherogenic metabolites such as the apolipoprotein B to A1 ratio (ApoB:ApoA1) are proposed to contribute to the sex-associated risk of cardiovascular disease (8). Many metabolite levels change with puberty, the menstrual cycle (9), pregnancy (10), oral contraceptive pill use (11), and with menopausal hormone replacement therapy (12), further supporting the notion that endogenous and exogenous sex hormones influence the metabolome. Feminizing GAHT is associated with elevated risk of cardiovascular disease including thrombosis, stroke, and myocardial infarction (13, 14), but its effect the plasma metabolome are unknown (15). Previous studies suggested that feminizing GAHT is associated with insulin resistance, but these effects may be related to the anti-androgen cyproterone acetate rather than estradiol, and recent data do not show an increased incidence of type 2 diabetes (16). The lipid profile in transgender individuals on GAHT appears to partly mirror that of the affirmed gender (17). Whether these changes impact cardiovascular risk requires further study.

A small, cross-sectional metabolomics study reported that young transgender women on early feminizing GAHT (puberty blockers and estradiol, n = 25) had increased levels of HDL-related metabolites and ApoA1 levels relative to postpubertal cisgender men (n = 15) (18). By contrast, young transgender men on early masculinizing GAHT (puberty blockers and testosterone, n = 26) had decreased levels of HDL measures and ApoA1 and increased very low-density lipoprotein (VLDL) measures and ApoB:ApoA1 relative to postpubertal cisgender women (n = 17) (18). There is a deficiency of longitudinal data, which are important for understanding time-dependent and individual-specific responses to hormonal change, both of which are critical in the era of personalized medicine. We previously showed that feminizing and masculinizing GAHT change the immune cell epigenetic profile of transgender individuals, mirroring epigenetic changes that occur during puberty (19). Longitudinal collection of biospecimens from transgender individuals on GAHT is a powerful way to investigate the molecular effects of GAHT and elucidate the role of estrogen and testosterone on clinically relevant biomarkers in circulation.

Here, we investigate longitudinal changes in plasma metabolic biomarkers during the first 6 months of feminizing GAHT in transgender women using nuclear magnetic resonance (NMR)-based metabolomics. In this randomized double-blind trial, we compare the metabolic biomarker trajectories of transgender women on estradiol in combination with 2 anti-androgens, cyproterone acetate or spironolactone. We also use NMR-based metabolomics data from an independent cohort of children and adults (CheckPoint Study) to contextualize GAHT-associated changes within sex- and age-related metabolite biomarker patterns.

## Materials and Methods

### Ethics and Recruitment

This research was conducted using biospecimens collected during the clinical trial titled “A randomized double-blind trial comparing the effectiveness of anti-androgen medications in trans and gender diverse individuals” (Universal Trial Number: U1111-1248-7232, ANZCTR registration ACTRN12620000339954) and was approved by the Austin Health Human Research Ethics Committee (Ethics Approval Number: HREC/44503/Austin-2018). This was a single-site study and participants were recruited at Austin Health (Heidelberg, Victoria, Australia) between August 31, 2020, and March 15, 2022. Inclusion criteria included transgender women aged 16 to 80 years who were about to commence treatment with feminizing GAHT with anti-androgens. Exclusion criteria included evidence of androgen deficiency before commencement of estradiol (total testosterone < 10 nmol/L), a planned orchiectomy within 6 months of joining the trial, or any contraindications to spironolactone, cyproterone acetate, or estradiol. Participants were provided with a Participant Information Sheet/Consent Form and gave informed consent for their blood samples to be used in future studies, including blood analysis and genetic analysis.

### Sample Size and Cohort Demographics

Plasma from 53 participants were used in this study. Plasma samples with moderate to gross hemolysis or inadequate volume for measurement of metabolic biomarkers were excluded. Mean age at the time of first blood collection was 25.9 years, with a range of 18 to 58 years. Cohort demographics including age, ethnicity, and body mass index (BMI) can be found in Table 1. Of the 53 individuals, 4 were underweight, 22 were healthy weight, 12 were overweight, and 15 were obese, and no participant changed “weight bracket” within the 6 months of GAHT. Additional comorbidities included depression/anxiety (20 participants), attention deficit hyperactivity disorder (8 participants), which did not change during the 6-month GAHT period.

**Table 1.** Transgender women cohort demographics and median sex hormone levels

	Spironolactone group	Cyproterone acetate group	Combined
Sample size (%)	26 (49.1)	27 (50.9)	53
Median age at baseline (range, IQR)	25 (20-58, 8)	24.5 (18-36, 7)	25.0 (18-58, 7.5)
Mean BMI kg/m^2^ (±SD)			
Baseline	27.0 (±6.9)	26.9 (±7.0)	27 (±6.9)
6 months’ GAHT	28.5 (±7.0)	27.1 (±6.15)	27.7 (±6.5)
Ethnicity (note that participants were able to select >1 ethnicity, so totals can exceed 100%)			
White	22	26	48 (90.6%)
Asian	3	1	4 (7.5%)
Black	2	0	2 (3.8%)
Aboriginal and Torres Strait Islander	1	0	1 (1.9%)
Median sex hormone levels			
Estradiol (pmol/L)			
Baseline	161.5	115.0	119.5
3 months’ GAHT	367.7	336.5	352.1
6 months’ GAHT	300.5	490.0	371.0
Total testosterone (nmol/L)			
Baseline	14.2	17.3	16.5
3 months’ GAHT	4.9	0.7	1.1
6 months’ GAHT	1.8	0.8	0.9
FSH (IU/L)			
Baseline	3.0	3.1	3.1
3 months’ GAHT	1.3	0.2	0.3
6 months’ GAHT	0.7	0.2	0.5
LH (IU/L)			
Baseline	4.2	4.3	4.3
3 months’ GAHT	2.7	0.2	0.7
6 months’ GAHT	1.8	0.2	0.5
Prolactin (mIU/L)			
Baseline	210.0	210.5	210.0
3 months’ GAHT	243.5	546.0	368.0
6 months’ GAHT	252.5	539.0	420.0
SHBG (nmol/L)			
Baseline	30.5	24.5	27.0
3 months’ GAHT	43.0	36.0	41.0
6 months’ GAHT	40.5	42.0	41.5

Abbreviations: BMI, body mass index; GAHT, gender-affirming hormone therapy; IQR, interquartile range.

### Estradiol Feminizing GAHT Regimen

All participants in the study (n = 53) underwent feminizing GAHT. Estradiol was typically initiated in the form of 2- to 4-mg oral estradiol valerate (tablet) or in transdermal equivalent (estradiol patches or gel). In line with Australian guidelines (20), serum estradiol and total testosterone levels were monitored, and the estradiol dose was adjusted monthly in the initial 3 months to maintain serum estradiol levels in the target range of 250 to 600 pmol/L (21).

### Anti-androgen Randomization and Regimen

In this randomized double-blind trial, participants were randomized using permuted block randomization to 1 of 2 anti-androgens: either 12.5 mg daily cyproterone acetate (n = 27) or 100 mg daily spironolactone (n = 26), in addition to their routine estradiol regimen. Most participants (n = 37) commenced anti-androgen and E2 therapy on the same day, and the remaining participants began anti-androgens within 6 weeks of commencing estradiol.

### Blood Collection and Plasma Sample Preparation

Approximately 5 mL of venous blood was collected for research in sodium heparin tubes at baseline (before GAHT administration) and after 3 and 6 months of GAHT. Blood was collected after an overnight fast. For all samples, the plasma component was separated from cellular components by centrifugation (10 minutes at 500*g*, room temperature). Plasma samples were aliquoted and stored at −80 °C until required for subsequent metabolomics.

### Sex Hormone Measurements

In line with Australian guidelines of GAHT clinical care (20), blood samples were taken monthly to monitor serum sex hormone levels before and during GAHT. Sex hormones included FSH, LH, estradiol, total testosterone, SHBG, and prolactin. National Association of Testing Authorities (the national accreditation body for Australia)-accredited laboratories available locally were used.

Serum estradiol and total testosterone were measured using liquid chromatography tandem mass spectrometry using the Shimadzu LC connected to an AB Sciex 5500 mass spectrometer. Testosterone was analyzed following addition of deuterated internal standard after simple liquid-liquid extraction using high-purity hexane/ethyl acetate, followed by injection into a C18 column connected to the electrospray source of the mass spectrometer. Quantitation used multiple reaction monitoring of 289.2/97.1 and 289.2/109.2 for confirmation with internal standard 292.2/97.3. The assay range was 0.1 to 34.0 nmol/L with precision of 4.8% at 0.4 and 2.4% at 12 nmol/L. Estradiol was analyzed following addition of deuterated internal standard, after simple liquid/liquid extraction into high-purity tertiary butyl methyl ether followed by injection into a C18 column connected to the electrospray source of the mass spectrometer. Quantitation used multiple reaction monitoring of 271.2/145.1 and 271.2/143.01 for confirmation with internal standard 274.1/145.0. Assay range was 10 to 18 000 pmol/L with precision of 20% at 14 and 9.8% at 51 pmol/L.

The remaining hormones (FSH, LH, prolactin, SHBG) were measured by electrochemiluminescence immunoassay (Beckman Coulter AU5800).

Median sex hormone levels at baseline and after 3 and 6 months of GAHT can be found in Table 1.

### NMR Metabolomics

Plasma samples were thawed and centrifuged (500*g*, 10 minutes), and a 350-µL aliquot was sent to Nightingale Health (Helsinki, Finland) for measurement of 249 metabolic biomarkers using high-throughput proton NMR (measured using the 2020 Nightingale Health bioinformatics protocol). Plasma samples were randomized within and across plates. The Nightingale Health protocol includes measurements of lipoprotein total concentration, particle size, concentrations of cholesterol, free cholesterol, esterified cholesterol, triglycerides, total lipids, as well as several relative concentrations. Lipoprotein subclasses include several densities and sizes: HDL (small, medium, large, very large), LDL (small, medium, large), intermediate-density lipoprotein, and VLDL (very small, small, medium, large, very large, and chylomicrons and extremely large) (22, 23). In addition to lipoproteins, the Nightingale Health platform covers some amino acids, apolipoproteins, other lipids, fatty acids, glycolysis-related metabolic biomarkers, ketone bodies, fluid balance measures, and inflammation measures (22, 23). Mean raw metabolic biomarker measures for each timepoint and anti-androgen group can be found in Supplementary Table S1 (24).

### Statistical Analysis of Plasma Metabolic Biomarkers

Raw metabolic biomarker measures were natural log transformed and internally standardized (*z*-score) before statistical analysis. All statistical analyses were performed in R version 4.1.2 (25). For mixed linear models, the “nlme” package was used (26). We were interested in the effect of several exposures on plasma metabolic biomarkers (the outcomes in these models):

1. Time on feminizing GAHT
2. Type of anti-androgen administered (spironolactone vs cyproterone acetate) at each timepoint
3. Circulating hormone levels (estradiol, total testosterone, FSH, LH, prolactin, and SHBG)

In linear models 1 through 3, age and baseline BMI were adjusted for as covariates. The linear models are discussed in more detail later. Model (1): To assess the effect of feminizing GAHT over time on metabolic biomarkers, we investigated differences in metabolic biomarker levels at 3 months GAHT relative to baseline, 6 months GAHT relative to baseline, and 6 months GAHT relative to 3 months GAHT. These models were stratified based on anti-androgen randomization group. A random intercept was included for participant ID. Model (2): To assess the effect of anti-androgen allocation on metabolic biomarkers, we ran linear models with data from each timepoint (baseline, 3 months, and 6 months), with anti-androgen allocation (spironolactone or cyproterone acetate) as a binary exposure. Model (3): To assess the association of circulating sex hormone levels with metabolic biomarkers, we ran linear models with each natural log-transformed hormone level (estradiol, total testosterone, FSH, LH, prolactin, or SHBG) as the exposure. A random intercept for participant identification was included. Within each model, *P* values were then corrected for false discovery rate using the Benjamini-Hochberg approach (27). Adjusted *P* values < .05 were considered significant.

### Substudy: Population-based Metabolomics Datasets

There is evidence of sexual dimorphism in circulating metabolite levels. Some metabolites remain sex-associated across the lifespan, whereas other become sex-specific during puberty, thought to be driven at least in part by sex hormones (termed “sex- and age-related” metabolites). To place our findings within this context, we used the Child Health CheckPoint Study (described in detail by (28)), part of the population-derived Longitudinal Study of Australian Children, as a substudy to investigate sex-associated metabolic biomarkers in children (aged 11-12 years) and adults (aged 28-71 years). The CheckPoint Study is a cross-sectional study of Australian children (aged 11-13 years, n = 919 assigned females and n = 955 assigned males) and their parents (aged 28-71 years, n = 1644 assigned females and n = 230 assigned males). Considering that there is a genetic component to circulating plasma metabolite levels (29), using a cohort with matched parent-child samples would mean that the change in metabolite levels over time are less likely to be genetically driven, but rather the result of hormone changes. The following data were provided: sex, age, BMI, and 228 serum metabolic biomarker levels (measured using the 2016 Nightingale Health bioinformatics protocol), in addition to the Sexual Maturity Rating (Tanner stage) self-reported from child participants as an estimate of pubertal development. Data were available from 1874 adults (parents of the child participants), of which 1644 (87.7%) were assigned female at birth and 210 were (11.3%) assigned male at birth, and from 1874 children, of which 919 (49%) were female and 955 (51%) were male.

The CheckPoint data were generated with an earlier version of the Nightingale platform (2016 version) compared with the feminizing GAHT cohort (2020 version). Although there are differences between the platforms, our top hits, glutamine and ApoB:ApoA1 ratio, are present in both and show a strong correlation (>0.9) between the 2 versions (30). Nightingale Health metabolic biomarker data from The CheckPoint Study were normalized and standardized as described previously. The CheckPoint Study dataset included zeros, so the minimum detectable reading for each metabolic biomarker was added to all CheckPoint Study samples to allow for natural log transformation, as previously described (31). We investigated sex-associated metabolic biomarkers in children and adults separately using linear regression models. In both child and adult linear models, assigned sex was the exposure, metabolic biomarker measures were the outcomes, and age and BMI were adjusted for as covariates. *P* values obtained from each model output were corrected for false discovery rate using the Benjamini-Hochberg correction. Adjusted *P* values of < .05 were considered statistically significant.

## Results

### Influence of Different Anti-androgens on Sex Hormone Dynamics During the First 6 Months of Feminizing GAHT

**Figure 1.**
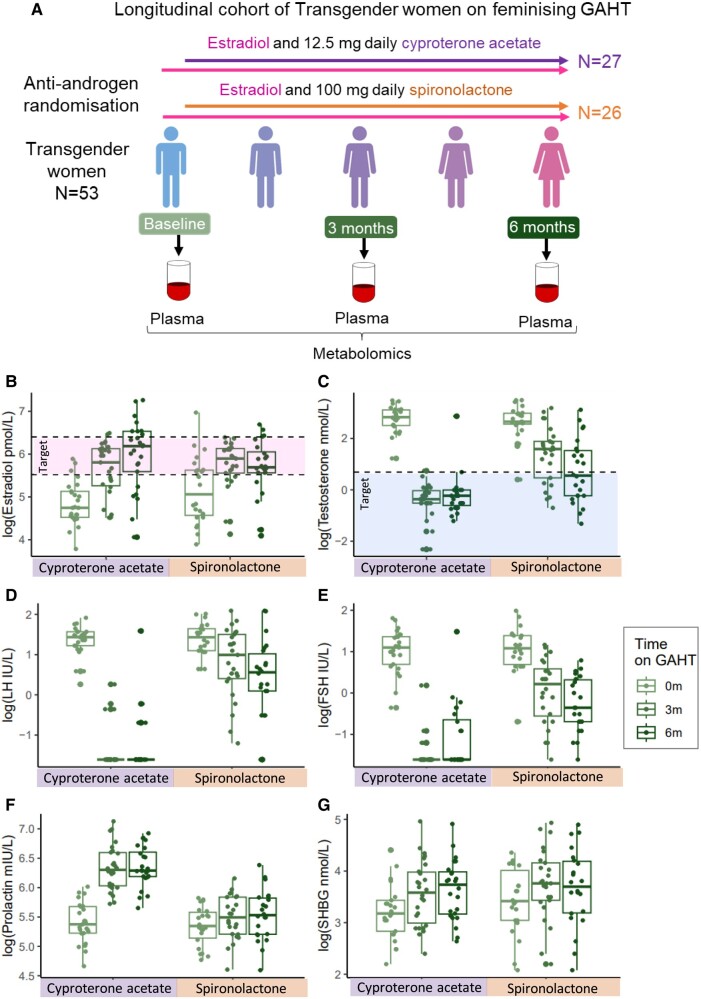
Changes to the circulating sex hormone milieu occur earlier and more potently in the cyproterone acetate group. (A) Study overview. N = 53 transgender women were recruited and randomly allocated to estradiol and 100 mg/day spironolactone or estradiol and 12.5 mg/day cyproterone acetate. Blood was collected at baseline (GAHT-naïve) and after 3 and 6 months. Heparinized plasma was sent for high-throughput NMR-based metabolomics. (B-G) Boxplots describing natural log-transformed hormone levels across GAHT in each anti-androgen group (baseline = 0 m, 3 months GAHT = 3 m, 6 months GAHT = 6 m): (B) estradiol, (C) total testosterone, (D) LH, (E) FSH, (F) prolactin, and (G) SHBG.

We collected blood at baseline and 3 and 6 months after feminizing GAHT from participants in a double-blind trial of 2 different anti-androgens, cyproterone acetate (n = 27) or spironolactone (n = 26), in combination with estradiol (Fig. 1A and Table 1). The participants ranged in age from 18 to 58 years at baseline. Median sex hormone levels within each anti-androgen group at each timepoint can be found in Table 1. Feminizing GAHT resulted in increased levels of circulating estradiol to a similar level in both groups, reaching target range (250-600 pmol/L) as early as 3 months and maintained or further increased by 6 months (Fig. 1B and Table 1). Circulating total testosterone was reduced over time in both anti-androgen groups; however cyproterone acetate had a more potent effect, lowering testosterone levels to within target range (<2 nmol/L) by 3 months (Fig. 1C and Table 1). In the cyproterone acetate group, 95.6% of transgender women had total testosterone levels in target range after 6 months compared with 54.1% in the spironolactone group. Similarly, cyproterone acetate had a more potent effect on lowering LH and FSH (Fig. 1D-1E, and Table 1) and a uniquely raised prolactin levels (Fig. 1F and Table 1).

### Feminizing GAHT Decreases Circulating Atherogenic Markers Over Time in Both Anti-androgen Groups

To assess the effect of estradiol and anti-androgens over time, we performed mixed linear regression models comparing each timepoint (3-month GAHT or 6-month GAHT) to baseline (pre-GAHT). The analysis was stratified by anti-androgen randomization group to identify metabolic biomarker trajectories unique to each anti-androgen.

**Figure 2.**
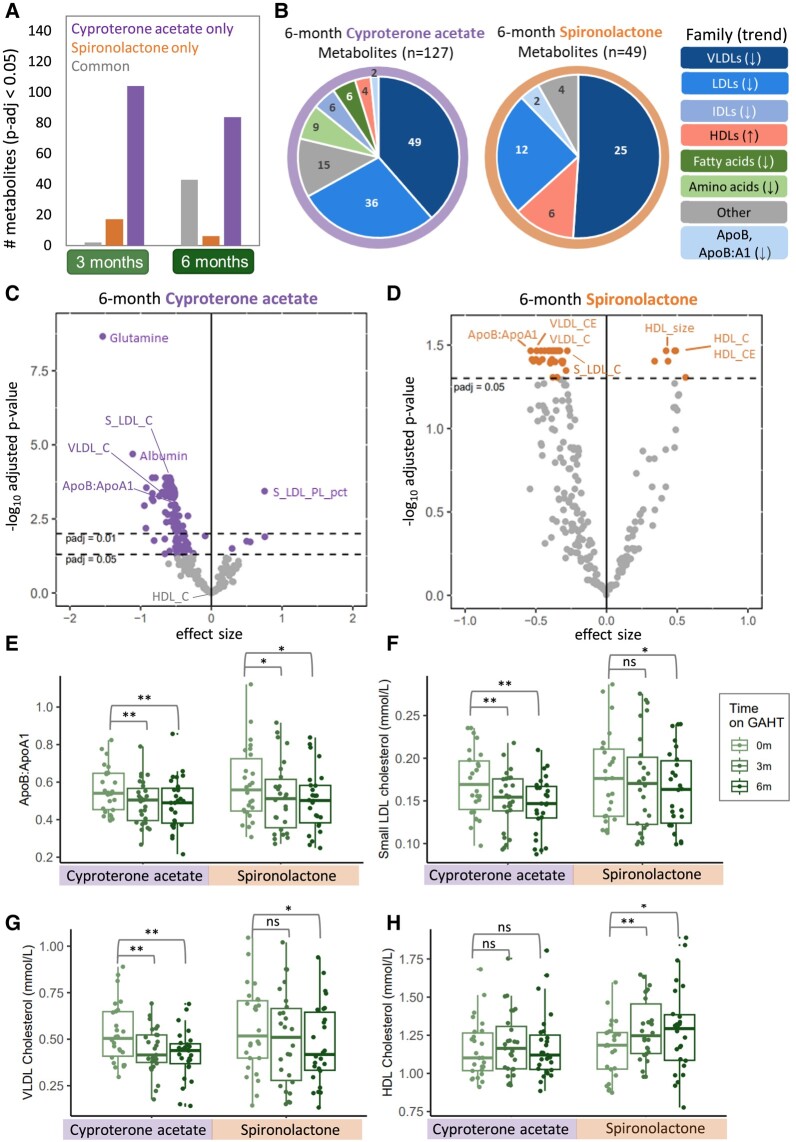
(A) Bar plot summarizing number of significant (adjusted *P* < .05) metabolic biomarkers associated with 3 or 6 months of GAHT relative to baseline. Purple bars indicate metabolic biomarkers only significant in the cyproterone acetate group, orange bars indicate metabolic biomarkers only significant in the spironolactone group, and gray bars indicating common metabolic biomarkers (significant in both anti-androgen groups). (B) Pie chart depicting proportions and trend (increase or decrease) of metabolic biomarker classes significantly altered after 6 months of GAHT relative to baseline in cyproterone acetate (left) and spironolactone (right) groups. In both groups, we observed decreased levels of VLDL, LDL, ApoB, and ApoB:ApoA1 ratio. (C-D) Volcano plots showing metabolic biomarkers significantly altered after 6 months of GAHT relative to baseline in cyproterone acetate (C) and spironolactone (D) groups. The y-axis represents –log10 Benjamini-Hochberg adjusted *P* value and the x-axis represents the effect size (beta coefficient) derived from the mixed linear model. The dashed y-intercepts indicate an adjusted *P* value cutoff of <.05 (bottom) or <.01 (top). (E-H) Boxplots describing metabolic biomarker levels across GAHT in each anti-androgen group (baseline = 0 m, 3 months of GAHT = 3 m, 6 months of GAHT = 6 m): (E) ApoB:ApoA1 ratio, (F) cholesterol concentration (mmol/L) of small low-density lipoprotein (LDL), (G) cholesterol concentration (mmol/L) of total very low-density lipoprotein VLDL, (H) cholesterol concentration (mmol/L) of total high-density lipoprotein (HDL). * Benjamini-Hochberg adjusted *P* value from timepoint mixed linear model < .05, ** Benjamini-Hochberg adjusted *P* value from timepoint mixed linear model < .01.

We identified 123 metabolic biomarkers as significantly altered (adjusted *P* < .05) after 3 months of GAHT relative to baseline, with 104 (84.6%) metabolic biomarkers unique to the cyproterone acetate group, 17 (13.8%) unique to the spironolactone group, and 2 (1.6%) altered in both groups (Fig. 2A and Supplementary Table S2 (24)). We identified 133 metabolic biomarkers significantly altered after 6 months GAHT relative to baseline, with 84 (63.1%) unique to the cyproterone acetate group, 6 (4.5%) unique to the spironolactone group, and 43 (32.3%) altered in both groups (Fig. 2A, Supplementary Table S2 (24)).

Of the 43 metabolic biomarkers altered after 6 months GAHT in both anti-androgen groups, 25 (58.1%) belonged to the VLDL family (including the concentration of small, medium, and total VLDL, and the cholesterol levels and esterified cholesterol levels of small, medium, large, extra-large, and total VLDL), and 12 (27.9%) belonged to the LDL family (including the concentration of small, medium, large, and total LDL, and cholesterol levels and esterified cholesterol levels of small and medium LDL), with all but 1 showing a decrease relative to baseline (Fig. 2B and Supplementary Table S2 (24)). The ApoB:ApoA1 ratio was significantly decreased after both 3- and 6-month GAHT relative to baseline in both anti-androgen groups (Fig. 2C-2E and Supplementary Table S2 (24)). We included longitudinal plasma measurements for a cisgender male as a technical control, which showed stability in the ApoB:ApoA1 ratio over time (Supplementary Fig. S1C and S1E (24)). We observed larger effect sizes and smaller *P* values in the cyproterone acetate timepoint models compared with spironolactone for VLDL, LDL, and apolipoprotein metabolic biomarkers (Fig. 2C, 2D, 2F, and 2G). Together, these results show that 6 months of GAHT induces a significant decrease in plasma atherogenic markers in both anti-androgen groups, with cyproterone acetate having a more pronounced effect on commonly affected lipoprotein and apolipoprotein measures.

Total cholesterol, total free cholesterol, and total esterified cholesterol levels were significantly decreased after 3 and 6 months in the cyproterone acetate group, but not in the spironolactone group (Supplementary Table S2 (24)).

In the spironolactone group alone, we observed significantly increased plasma total HDL measures (including total HDL cholesterol concentration [Fig. 2H], total HDL cholesterol ester concentration, total HDL free cholesterol concentration, and total HDL particle diameter) at the 3-month timepoint relative to baseline; this effect was maintained at the 6-month timepoint (Fig. 2D, Supplementary Table S2 (24)). These results indicate that spironolactone in combination with estradiol has an early and unique effect on raising HDL measures, which may also offer a further beneficial shift in the plasma metabolome, in addition to reduction in VLDL, LDL, and ApoB measures.

### Cyproterone Acetate Administration Results in Decreased Levels of Certain Plasma Amino Acids

**Figure 3.**
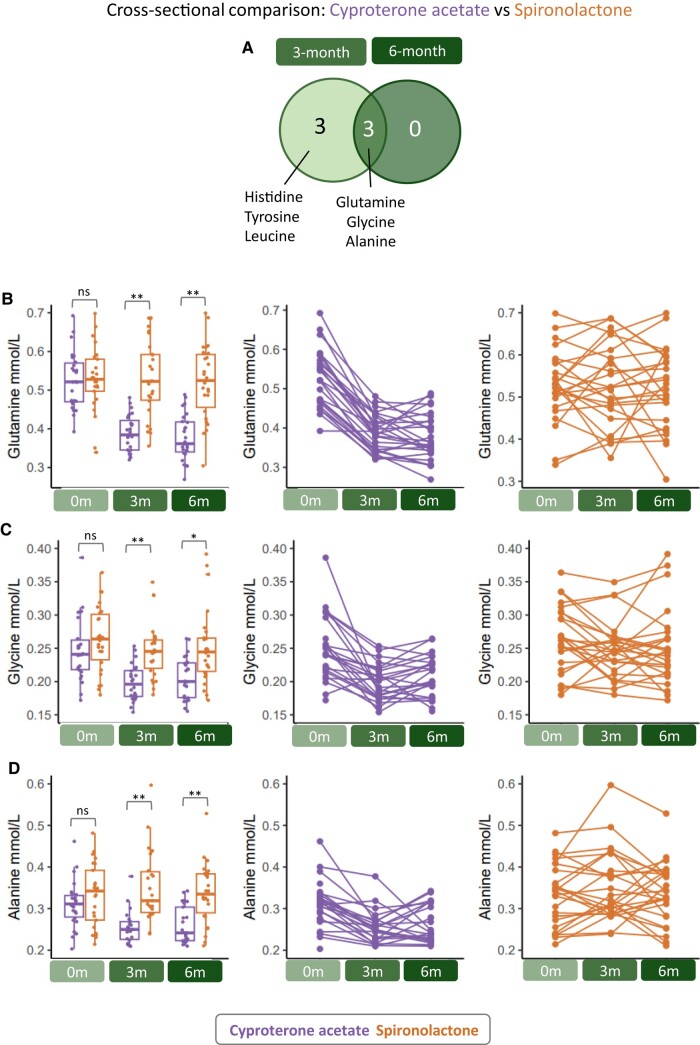
Unique changes in the amino acid profile after 3 and 6 months of GAHT in the cyproterone acetate group. (A) Cross-sectional analysis of spironolactone vs cyproterone acetate groups at 3 and 6 months of GAHT revealed amino acids significantly (Benjamini-Hochberg adjusted *P* < .05) different at 3 months or 6 months (or both). (B-D) Boxplots showing glutamine (B), glycine (C), and alanine (D) (mmol/L) levels in the cyproterone acetate (orange) and spironolactone (groups) at baseline, 3 months, and 6 months of GAHT. Line graphs showing individual participant trajectories of glutamine (B), glycine (C), and alanine (D) (mmol/L) across GAHT in the cyproterone acetate (purple) and spironolactone (orange) groups. *Benjamini-Hochberg adjusted *P* value from cross-sectional analysis linear model < .05, **Benjamini-Hochberg adjusted *P* value from cross-sectional analysis linear model < .01.

To further investigate differences between anti-androgen groups, we analyzed metabolite biomarker levels cross-sectionally to identify significant (adjusted *P* < .05) differences between groups at baseline, and after 3 and 6 months of GAHT (Fig. 3A and Supplementary Table S2 (24)). As expected, no significant differences between anti-androgen groups were observed at baseline.

At the 3-month timepoint, we observed significantly lower levels of amino acids glutamine (Fig. 3B), glycine (Fig. 3C), alanine (Fig. 3D), histidine (Supplementary Fig. S2B (24)), tyrosine (Supplementary Fig. S2C (24)), and leucine (Supplementary Fig. S2D (24)) in the cyproterone acetate group compared with spironolactone group. At the 6-month timepoint, glutamine, glycine, and alanine remained significantly lower in the cyproterone acetate group compared with spironolactone with the same magnitude of difference as the 3-month timepoint (Fig. 3B-3D), suggesting that the cyproterone acetate-associated reduction in these amino acids occurs early and is maintained. Glutamine was also the most significantly altered metabolic biomarker in the cyproterone acetate timepoint comparison (Fig. 2C and Supplementary Table S2 (24)) as well as the cross-sectional comparisons (Fig. 3B and Supplementary Table S2 (24)). We included a longitudinal cisgender male sample as a technical control that showed stability over time in glutamine levels (Supplementary Fig. S1D and S1F (24)). No significant changes in amino acids were detected over time in the spironolactone group, highlighting that unique effect of cyproterone acetate on the plasma amino acid profile.

### GAHT-affected Metabolic Biomarkers are Associated With Circulating sex Hormone Levels

**Figure 4.**
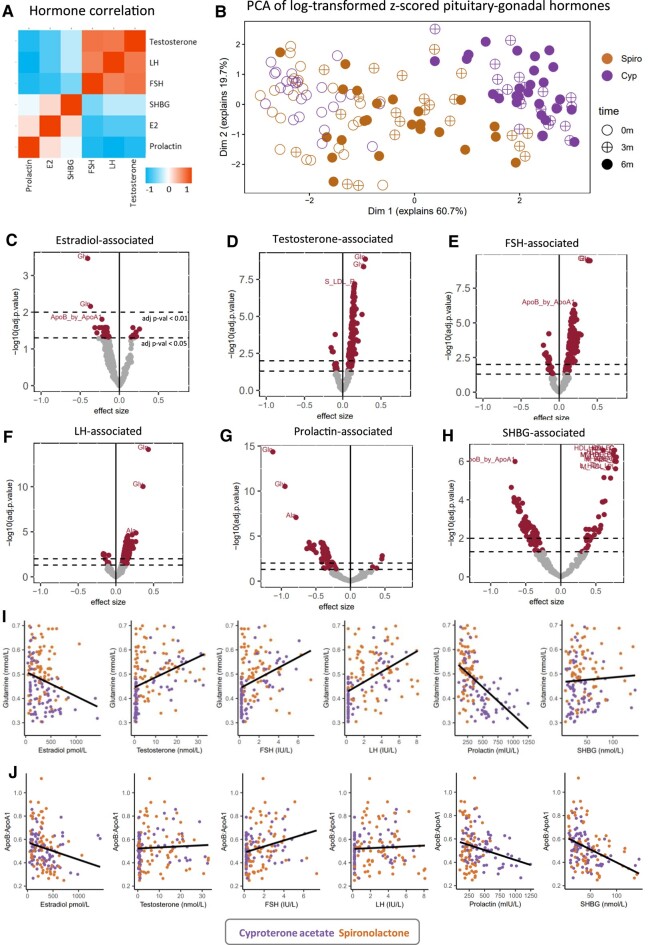
Plasma metabolic biomarker levels are significantly associated with circulating hormone levels in transgender women. (A) Pearson's correlation plot of log-transformed and *z*-scored circulating sex hormones (estradiol, total testosterone, FSH, LH, prolactin, and SHBG). (B). Principal component analysis (PCA) plot of natural log transformed *z*-scored sex hormone levels (estradiol, total testosterone, FSH, LH, prolactin, and SHBG). Cyproterone acetate group samples show better separation from baseline samples than spironolactone samples on PC1 (dimension 1, x-axis), indicative of a more pronounced change in the hormonal milieu from baseline. (C-H) Volcano plots showing metabolic biomarkers significantly associated with circulating levels of estradiol (C), total testosterone (D), FSH (E), LH (F), prolactin (G), and SHBG (H). The y-axis represents –log10 Benjamini-Hochberg adjusted *P* value and the x-axis represents the effect size (beta coefficient) derived from the mixed linear model. The dashed y-intercepts indicate an adjusted *P* value cutoff of <.05 (bottom) or <.01 (top). Metabolic biomarkers highlighted in maroon indicate those with an adjusted *P* value < .05. Most significant metabolic biomarkers are annotated, with glutamine (Gln), glycine (Gly), alanine (Ala), and the ApoB:ApoA1 ratio (ApoB_by_ApoA1) commonly among top metabolic biomarkers. (I-J) Scatter plots showing the association of glutamine (mmol/L) (I) and ApoB:ApoA1 ratio (mmol/L) (J) with circulating sex hormone levels. In (I-J), orange data points indicate those from the spironolactone group and purple data points indicate those from the cyproterone acetate group.

Considering the profound changes to the sex hormone milieu (Fig. 1) and to the plasma metabolome with feminizing GAHT (Fig. 2), we investigated the association of each circulating sex hormone (estradiol, total testosterone, FSH, LH, prolactin, and SHBG) with metabolic biomarkers (Fig. 4). Sex hormones correlate strongly with each other (Fig. 4A). The potent effect of cyproterone acetate on sex hormone levels over time (as shown in Fig. 1) is clear when visualizing samples in a principal component analysis plot based on sex hormone levels, with 3- and 6-month cyproterone acetate samples clustering further from baseline than 3- and 6-month spironolactone samples on dimension 1 (x-axis) (Fig. 4B). All hormones (estradiol, total testosterone, FSH, LH, prolactin, and SHBG) were significantly associated (adjusted *P* < .05) with numerous plasma metabolic biomarkers, with FSH levels associated with the most (152 metabolic biomarkers) (Supplementary Table S3 (24)) and estradiol associated with the least (68 metabolic biomarkers). All significant hormone-metabolic biomarker associations are reported in Supplementary Table S3 (24). Nearly all (96%) of GAHT-associated metabolic biomarkers were significantly associated with at least 1 sex hormone, suggesting an interplay between the changes in the sex hormone milieu and changes in the plasma metabolome across feminizing GAHT.

In terms of glutamine, glycine, and alanine—the amino acids uniquely in the cyproterone acetate group—prolactin showed the strongest negative association (Fig. 4G and 4I), and LH showed the strongest positive association (Fig. 4F and 4I) with these amino acids. This is consistent with these hormones uniquely changing over time with cyproterone acetate but not spironolactone (Fig. 1D and 1F), suggesting a relationship between cyproterone acetate, sex hormones (particularly prolactin, FSH, and LH), and these specific plasma amino acids. In line with this, we see a positive correlation of glutamine with total testosterone, FSH, and LH, and a negative correlation with estradiol and prolactin (Fig. 4I).

Considering that hormones correlate with each other (Fig. 4A), it is therefore unsurprising that numerous metabolic biomarkers were significantly associated with more than 1 sex hormone. For example, total testosterone and estradiol levels are negatively correlated in our cohort (Fig. 4A), and we found that all metabolic biomarkers associated with both estradiol and total testosterone (n = 67) had opposing directions of associations (Supplementary Fig. S3D (24)). Glutamine, glycine, alanine, and ApoB:ApoA1 were among metabolic biomarkers showing the strongest evidence of association with sex hormones (Fig. 4C-4H and 4I-4J, and Supplementary Table S3 (24)). Together, our results reveal that nearly all GAHT-affected metabolite biomarkers are associated with sex hormones. We also show that the unique amino acid changes in the cyproterone acetate group are significantly associated with the unique cyproterone acetate-induced changes in the sex hormone milieu.

### Feminizing GAHT Alters Sex- and Age-related Metabolic Biomarkers Toward the Profile of Those Assigned Female at Birth

**Figure 5.**
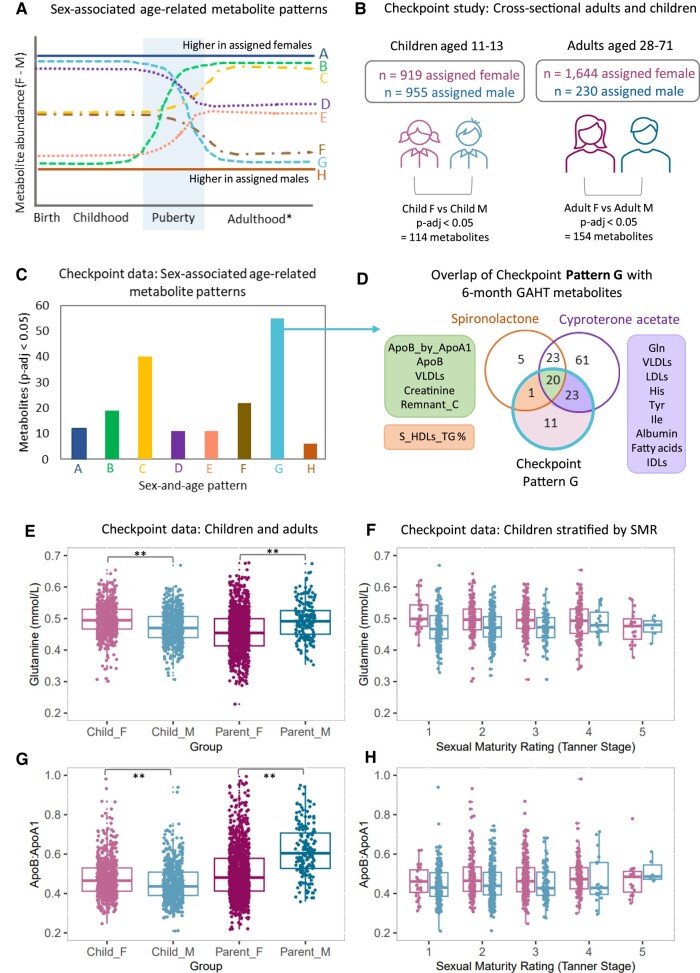
Top GAHT-affected metabolic biomarkers are sex- and age-associated metabolic biomarkers. (A) Circulating metabolic biomarkers show sex-associated age-related patterns. We identify 8 patterns (“A” to “H”), 2 of which are sex-associated across the lifespan (“A” and “H”) and 6 of which show sex- and age-related patterns (“B” to “G”). B. We used Nightingale Health metabolomics data from the CheckPoint to identify significant (adjusted *P* < .05) sex-associated metabolic biomarkers in children and adults. (C) Patterns of sex-associated metabolic biomarkers in CheckPoint data, based on the 8 patterns (“A”-“G”). Pattern “G” metabolic biomarkers (sex-associated in children and adults, with higher levels in female children and lower levels in female adults) was the most common pattern. (D) Overlap of pattern G metabolic biomarkers identified in the CheckPoint study with 6-month GAHT-affected metabolic biomarkers. Forty-four of 55 (80%) were significantly altered after 6 months of GAHT, with 23 unique to the cyproterone acetate group, 1 metabolic biomarker specific to the spironolactone group, and 20 common metabolic biomarkers. (E-F) Boxplots showing glutamine (mmol/L) levels grouped by age (child or adult) and sex (pink = assigned female, blue = assigned male) (E) and Sexual Maturity Rating (Tanner stage) in children (F). (G-H) Boxplots showing the ApoB:ApoA1 ratio grouped by age and sex (G) and Sexual Maturity Rating (Tanner stage) in children (H). **Benjamini-Hochberg adjusted *P* < .01. In (F) and (H), sex association appears to shift from child association to toward adult association as Sexual Maturity Rating category increases.

We hypothesize that GAHT-associated metabolic biomarker changes we report here are enriched for “sex- and age-related” metabolic biomarkers and will show a shift toward an assigned female level (Fig. 5A). For details on all “sex- and age-related patterns” shown in Fig. 5A, please see Supplementary Table S4 (24).

To address these hypotheses, we used Nightingale Health serum metabolomics data from the CheckPoint Study (28) (Fig. 5B). We identify 114 sex-associated metabolic biomarkers in children and 154 in adults (Fig. 5B and Supplementary Table S4 (24)). To identify sex- and age-related metabolic biomarkers in the CheckPoint Study, we overlapped sex-associated metabolic biomarkers in children and adults. We identified 8 patterns (A to H), 2 are sex-associated across the lifespan (A, H) and 6 show sex- and age-related patterns (B-G) (Fig. 5A and Supplementary Table S4 (24)). The most abundant metabolic biomarker pattern (pattern G) shows higher levels in children assigned female and lower levels in adults assigned female, compared with assigned males (n = 55 metabolic biomarkers) (Fig. 5A and 5C). Of these 55 metabolic biomarkers, 80% (44/55) were significantly lowered after 6 months of feminizing GAHT (Fig. 5D). In particular, our top 2 hits, glutamine and ApoB:ApoA1, were higher in female children compared with male children but lower in female adults compared with male adults (Fig. 5E). The same is seen when stratifying by the Sexual Maturity Rating in child participants, with both glutamine and ApoB:ApoA1 decreasing as puberty progresses (Fig. 5E and 5F), further supporting a role of sex hormones in sex-associated metabolic biomarker levels.

## Discussion

This study is the first to characterize the longitudinal plasma metabolome across the first 6 months of feminizing GAHT in transgender women, and to compare the effect of 2 common anti-androgens, spironolactone and cyproterone acetate, administered in combination with estradiol. Overall, we observed a shift in plasma metabolome toward the female profile in both groups, which was more evident in the cyproterone acetate group. There was a significant reduction in metabolic biomarkers belonging to VLDL and LDL family as well as lowering of the ApoB:ApoA1 ratio over 6 months of GAHT in both anti-androgen groups, which may be indicative of a beneficial cardiovascular profile. We observed reduction in the plasma amino acids glutamine, glycine, and alanine in the cyproterone group only. The relevance of this for the health of transgender women requires further research, but glutamine and alanine levels are associated with type 2 diabetes risk (32). Additionally, elevated alanine levels are associated with incidence of cardiovascular disease (33), indicating a protective effect in feminizing GAHT.

Estradiol has long been considered antiatherogenic, with adult females having a beneficial lipid profile compared with age-matched males (18). In line with this, adult females have a reduced atherosclerotic cardiovascular disease risk (18). However, studies investigating the effect of feminizing GAHT on cardiovascular disease risk and on biomarkers of cardiovascular disease are inconsistent. As with other exogenous estradiol therapies (such as the oral contraceptive pill and menopausal hormone therapy), venous thromboembolism is a rare but serious side effect of estradiol feminizing GAHT (34). In large medical record studies, incidence and risk of myocardial infarction and stroke are higher in transgender women compared to cisgender women, but results comparing transgender women to cisgender men are conflicting (13, 14, 34). It remains unclear whether specific anti-androgens influence cardiovascular disease risk.

We observed unique and common effects of spironolactone and cyproterone acetate on the plasma metabolic profile, with enrichment of sex- and age-related metabolic biomarkers that show a shift toward the female profile. No studies to date have captured the longitudinal changes in lipids and lipid subclasses across estradiol and anti-androgen therapy at the depth provided by high throughput NMR-based plasma metabolomics. In both the cyproterone acetate group and the spironolactone group, we observed a reduction in numerous LDL and VLDL measures after six months. Reductions in total and subclass VLDL and LDL measures were typically more pronounced and occurred earlier in the cyproterone acetate group, suggesting a more potent effect compared to spironolactone when combined with estradiol. This largely aligns with previous literature, with studies reporting decreased serum LDL cholesterol levels (35-37), or no significant change (38) after estradiol and cyproterone acetate GAHT.

Sexual dimorphism in LDL cholesterol levels has been widely reported and is thought to be driven by estrogen, with adult premenopausal females having lower levels compared with age matched males and postmenopausal females (7). Indeed, we found that LDL and VLDL cholesterol levels were negatively associated with estradiol levels. Estradiol hormone replacement therapy in postmenopausal females decreases hepatic lipase levels and activity, which is thought to decelerate the conversion of intermediate density lipoprotein to LDL (39), thus reducing LDL levels. Animal and in vitro studies have also shown that estradiol increases hepatic LDL receptor expression, which is thought to accelerate excretion of circulating LDL cholesterol (reviewed in (40)). These may be mechanisms by which feminizing GAHT lowers LDL levels, and indeed, Elbers et al (35) reported reduced hepatic lipase levels in transgender women after 1 year of estradiol and cyproterone acetate GAHT. We previously show that feminizing GAHT induces a loss of methylation in a region of the *VMP1* gene in leukocytes (19), and this gene is implicated in lipoprotein secretion (41, 42). Together, our results show a significant decrease in numerous LDL and VLDL measures in transgender women on estradiol and anti-androgen GAHT, and this may represent a cardioprotective, antiatherogenic shift in the plasma lipidome.

To the best of our knowledge, we are the first to report longitudinal changes in circulating ApoB levels and changes to the ApoB:ApoA1 ratio across feminizing GAHT. The ApoB:ApoA1 ratio is an effective biomarker for predicting metabolic syndrome (43) and coronary artery disease independent of LDL and HDL cholesterol markers (44). The ApoB:ApoA1 ratio has been reported as sex-associated and age-related, with higher levels in male children (8 years), but lower levels in males at average age of 16, 18, 25, and 50 years compared with females (45). We found a similar sex-and-age pattern in the CheckPoint study data included in this study. Congruently, the optimal cutoff values for predicting metabolic syndrome and coronary artery disease in adults are also sex-specific, with males having a higher cutoff than females. Reference ranges are typically established in cisgender individuals, and it remains unclear whether measures from transgender individuals on GAHT should be interpreted using the cutoff of their assigned sex or affirmed gender. We showed that the plasma ApoB:ApoA1 ratio decreases as early as 3 months in transgender women on estradiol and anti-androgen GAHT and this is maintained by 6 months, with estradiol levels (and other hormone levels) showing a significant negative association. Longitudinal studies are required to determine whether this decrease translates to a clinically significant benefit.

We observed that plasma glutamine, glycine, and alanine levels were significantly lower in the cyproterone acetate group compared with the spironolactone group after 3 and 6 months of GAHT (Fig. 3). Of these, glutamine showed the strongest evidence for a reduction over time. Glutamine is the most abundant amino acid in the circulation and is important for regulating immune cell metabolism and function (46). Glutamine is the precursor to glutamate, which is the most abundant excitatory neurotransmitter and can be used for energy production (47). Glutamine levels have been previously reported as sex-associated and age-related, with higher levels in female children (48) and lower levels in female adults compared with males (48-51). Bell et al (45) found that circulating glutamine levels were higher in females compared with males aged 8 years, but lower in females compared with males at ages 16, 18, 25, and 50 years, further supporting the role of pubertal changes in driving sex differences. We observed a similar sex- and age-related pattern in the CheckPoint study. A study in mice reported a lower glutamine to glucose ratio (Gln/Glu) in males and showed that androgen modulates circulating glutamine levels and Gln/Glu ratio in mice partially via the gut microbiome, which affects insulin sensitivity (52). Whether sex differences in circulating glutamine levels meaningfully contributes to sexual dimorphism in metabolic disease or immunity remains unexplored. Glutamine, glycine, and alanine levels have also been reported to change across the menstrual cycle and with combined oral contraceptive use in females (53-55), suggesting an interplay between sex hormones and amino acid levels. Indeed, we report a significant association of glutamine, glycine, and alanine with circulating levels of several sex hormones, particularly those that are uniquely altered with cyproterone acetate (prolactin, FSH, and LH). Further research is required to identify the mechanism of action of cyproterone acetate and estradiol GAHT on pituitary-gonadal hormones and plasma amino acid levels. Cyproterone acetate was associated with higher serum prolactin concentrations than spironolactone, though this was less than twice the upper limit of normal. This is consistent with previous studies which have shown a reversible and dose-dependent effect of cyproterone acetate on serum prolactin (56). Notably, large observational studies have suggested an association with higher cumulative cyproterone acetate exposure and meningioma (57), prompting the European Medicines Agency Safety Committee to recommend using alternatives to cyproterone acetate where practicable and avoiding doses ≥10 mg daily (58). In Australia, cyproterone acetate is available commercially only in 50- or 100-mg strength tablets.

One limitation of the study is the lack of an estradiol-only GAHT group, which would have allowed us to identify the effects of estradiol in the absence of anti-androgens. Although the anti-androgen regimen was fixed, participants had tailored estradiol regimens with doses increasing or decreasing as the study progressed to keep estradiol levels in target range (in line with Australian guidelines). Considering the individualized nature of estradiol doses and formulations (transdermal vs oral), we used circulating estradiol levels as a more accurate measure of estradiol exposure at the time of blood collection. Nonetheless, the longitudinal aspect of our cohort allowed each participant to be compared with their own pre-GAHT metabolome, which is a strength of the study and increases the statistical power in an otherwise relatively small sample size. Additionally, the cohort of transwomen in this study was predominantly of white European ancestry, particularly in the cyproterone acetate group, and future studies in diverse populations are required to confirm the generalizability of our findings. Finally, the samples size in this manuscript was low (n = 53 participants), and future studies with larger cohorts are necessary to understand the level of interindividual variation in response to feminizing GAHT, and factors that influence these responses.

In summary, metabolic biomarkers altered by feminizing GAHT were enriched for those that become sex-specific across puberty, and largely show a shift away from the male profile toward the female profile. Both cyproterone acetate and spironolactone in combination with estradiol decreased levels of atherogenic markers (VLDL cholesterol, ApoB, and the ApoB:ApoA1 ratio, among others), indicating a beneficial shift in the metabolome, with cyproterone acetate generally having a faster and more potent effect. Spironolactone had a unique effect on raising total HDL measures, and cyproterone acetate had a unique effect on lowering glutamine, glycine, and alanine levels. Our finding that spironolactone increases HDL-cholesterol is in line with previous studies (59, 60); however in our cohort cyproterone acetate did not decrease HDL-cholesterol, as previously reported (36, 59). The stronger effect of cyproterone acetate in decreasing LDL is also in line with previous findings (36, 59). The stronger effect of cyproterone acetate on LDL could be explained by its progestogenic actions or direct blocking of the androgen receptor, neither of which is a property of spironolactone. However, more research is required to understand the mode of action, because a meta-analysis of progestins in menopausal hormone replacement therapy found that it might actually blunt the benefit of estrogen on reducing LDL (61). The changes in metabolome may confer potential benefits in reducing cardiovascular risk in transgender women on feminizing GAHT and given prior retrospective studies observing increased cardiovascular events, further prospective longitudinal research examining cardiovascular disease in transgender women who are using contemporary formulations of feminizing GAHT are required.

## Data Availability

Original data generated and analyzed during this study are included in this published article or in the data repositories listed in References.

## Clinical Trial Registration

ClinicalTrials.gov registration no. ACTRN12620000339954.

## Abbreviations

ApoA1: apolipoprotein A1
ApoB: apolipoprotein B
BMI: body mass index
GAHT: gender-affirming hormone therapy
HDL: high-density lipoprotein
LDL: low-density lipoprotein
NMR: nuclear magnetic resonance
VLDL: very low-density lipoprotein
